# Pylephlebitis Complicated by Bacteremia: A Rare Complication Following Laparoscopic Cholecystectomy

**DOI:** 10.7759/cureus.39440

**Published:** 2023-05-24

**Authors:** Jawad Noor, Ahtshamullah Chaudhry, Saima Batool

**Affiliations:** 1 Hospital Medicine, St. Dominic Hospital, Jackson, USA; 2 Internal Medicine, St. Dominic Hospital, Jackson, USA; 3 Pathology, Nishtar Medical University, Multan, PAK

**Keywords:** septic thrombophlebitis, cholecystectomy, portal vein thrombosis, bacteremia, pylephlebitis

## Abstract

Pylephlebitis is a rare but serious condition caused by intra-abdominal or pelvic infections that can lead to septic thrombophlebitis of the portal veins. While laparoscopic cholecystectomy is considered a safe and effective treatment option, it is not without its risks, and pylephlebitis following this procedure is an extremely rare occurrence. Here, we present the case of a 73-year-old male who presented with lower abdominal pain for the last two weeks. He had undergone laparoscopic cholecystectomy for symptomatic cholelithiasis four weeks prior with an unremarkable follow-up. Laboratory tests revealed leukocytosis and blood culture showed *Streptococcus constellatus*. A CT scan revealed portal vein thrombosis causing diffuse periportal edema throughout the liver. The patient was treated with antibiotics and anticoagulation for pylephlebitis.

## Introduction

Pylephlebitis is a rare but serious condition characterized by septic thrombophlebitis of the portal veins caused by an intra-abdominal or pelvic infection of any etiology, leading to thrombosis and subsequent inflammation. While typically caused by infections such as appendicitis, diverticulitis, or inflammatory bowel disease, pylephlebitis as a complication following laparoscopic cholecystectomy is an extremely rare occurrence [1]. Advances in imaging technology have aided in the earlier detection and diagnosis of this disease process, which before the advent of widespread antibiotic use was almost universally fatal.

Laparoscopic cholecystectomy is a commonly performed surgical procedure for the removal of the gallbladder [2]. It is considered a safe and effective treatment option, with a low incidence of complications. However, like any surgical procedure, laparoscopic cholecystectomy is not without its risks. Pylephlebitis can occur after cholecystectomy due to factors such as venous stasis from pneumoperitoneum, perforation of the gallbladder, or iatrogenic bile duct injury, though it is an extremely rare occurrence [2]. Here, we report a rare case of pylephlebitis complicated by bacteremia following laparoscopic cholecystectomy.

## Case presentation

A 73-year-old male with a past medical history of benign prostatic hyperplasia and hyperlipidemia presented to the emergency room with a complaint of lower abdominal pain for the last two weeks which progressively worsened over time. Two days before admission he started having nausea associated with subjective fever and chills. Four weeks before admission, he had undergone laparoscopic cholecystectomy for symptomatic cholelithiasis without any complication. His postoperative follow-up was unremarkable. On presentation, his vital signs showed a temperature of 98.8°F, pulse of 95 beats/minute, blood pressure of 111/70 mmHg, and oxygen saturation of 97% on room air.

The physical examination showed an alert and awake male in mild distress due to abdominal pain. The lungs were clear to auscultation bilaterally with no respiratory distress, and heart sounds were normal with a regular rate, regular rhythm, and no murmurs. The abdominal examination revealed lower abdominal diffuse tenderness without guarding or rigidity.

**Figure 1:**
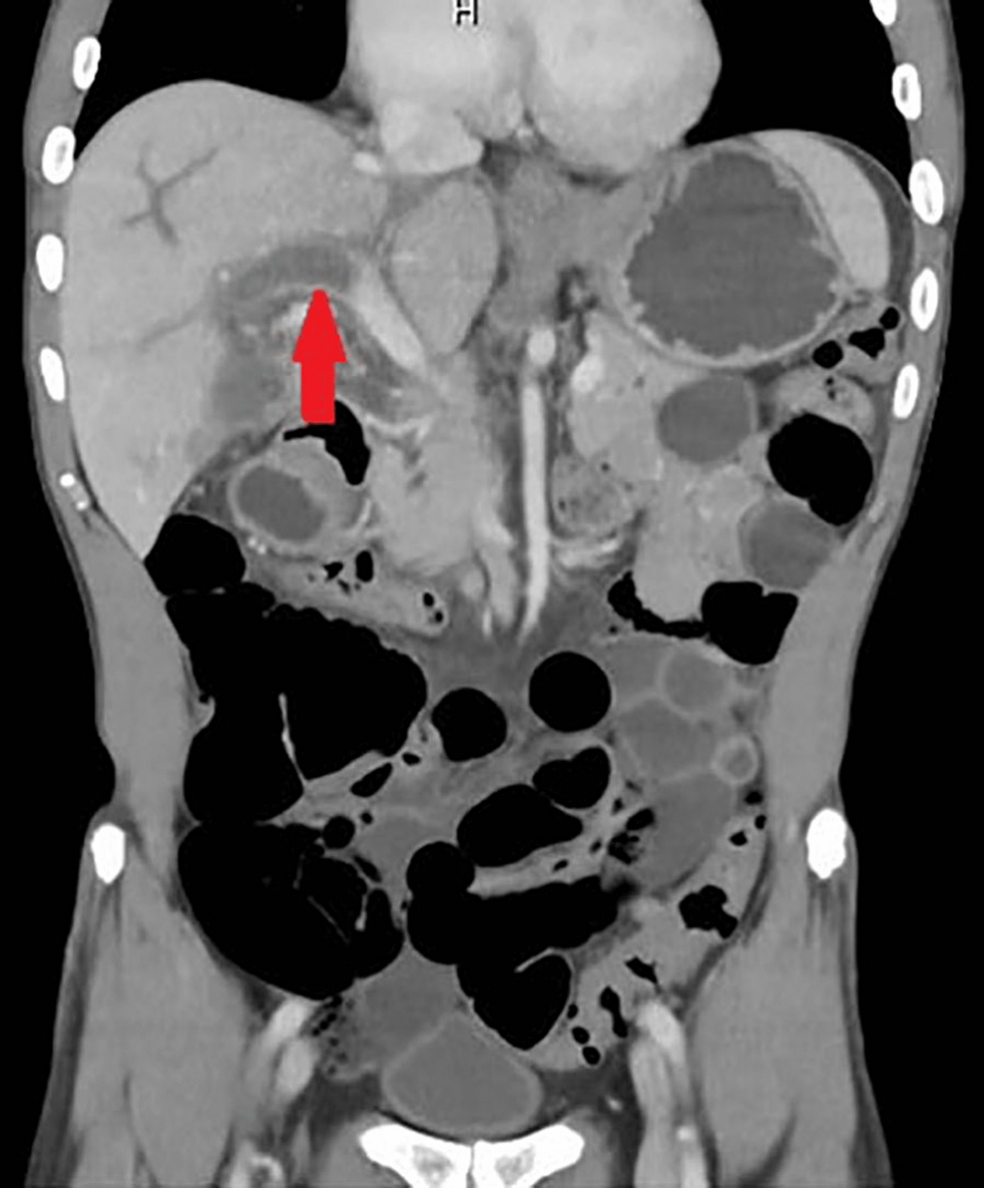
CT of the abdomen and pelvis pointing at portal vein thrombosis.

Labs were significant for leukocytosis with a white blood cell count of 18,200/µL, 88% neutrophils, and procalcitonin of 4.52 ng/mL. Blood culture was positive for *Streptococcus constellatus*. Chest X-ray showed no abnormality. CT of the abdomen and pelvis with intravenous contrast showed occlusion of the left and right main portal veins, favored to represent thrombus causing diffuse periportal edema throughout the liver, as shown in Figure 1.

**Figure 2:**
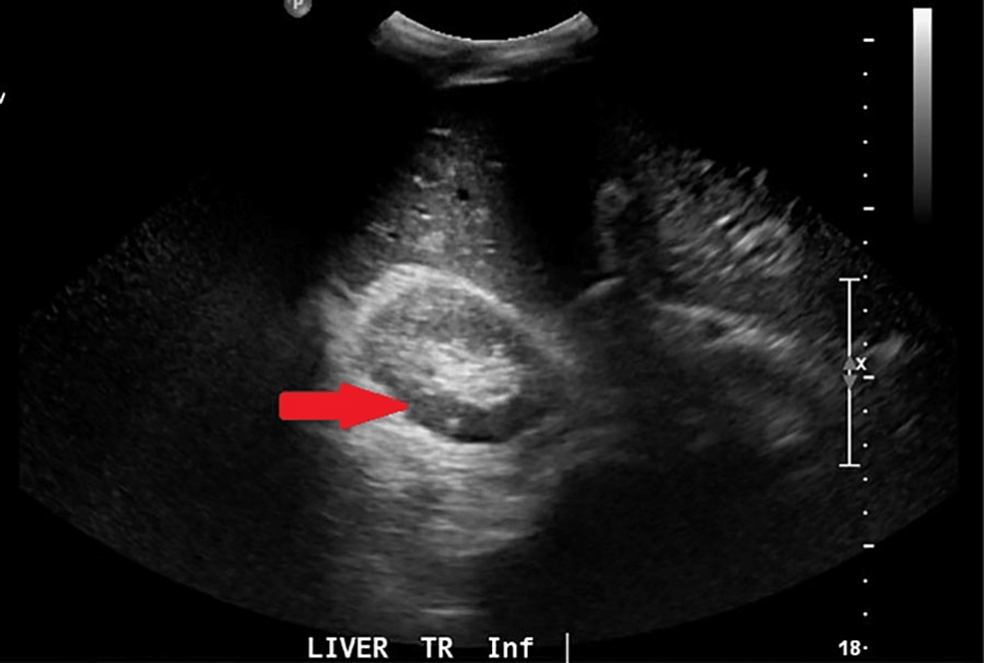
Ultrasound of the liver pointing to portal vein thrombosis.

Ultrasound of the abdomen revealed portal vein thrombosis with no evidence of cirrhosis, as shown in Figure 2.

Of note, an ultrasound of the abdomen done a few days before laparoscopic cholecystectomy showed no evidence of portal vein thrombosis.

The patient was started on a therapeutic dose of enoxaparin for portal vein thrombosis and on Rocephin for *Streptococcus *bacteremia. Further workup including CT of the chest, cancer antigen 19-9, prostate-specific antigen, and multiple myeloma workup was done to rule out malignancy as an underlying cause of portal vein thrombosis. All workup findings came back negative to suggest any underlying malignancy. Considering bacteremia and CT abdomen and pelvis findings of portal vein thrombosis with diffuse periportal edema throughout the liver, it was concluded that the patient had infected portal vein thrombosis which was a source of his bacteremia. After an improvement in his symptoms, he was discharged on intravenous Rocephin for a total of four weeks and apixaban.

## Discussion

Pylephlebitis is a rare condition that occurs when the portal venous system becomes infected and develops a thrombus, also known as suppurative thrombophlebitis. This is typically caused by intra-abdominal infections, such as diverticulitis or appendicitis [3]. The condition was first identified in 1846 during an autopsy on a patient with a hepatic abscess [4]. Although the incidence of pylephlebitis is not precisely known, it is estimated to occur in about 2.7 individuals per 100,000 annually [3].

It is typically caused by infections that affect the portal system and surrounding areas, beginning in smaller mesenteric veins and progressing to larger veins of the portal system and liver. It can also arise through bacterial seeding of pre-existing portal vein thrombosis [5]. Common causes include diverticulitis, appendicitis, pancreatitis, inflammatory bowel disease, and other abdominal infections. Certain procedures and risk factors such as hypercoagulable states or clotting factor deficiencies, recent abdominal surgery, malignancy, smoking, steroid use, and immobility can also trigger the condition [6].

Pylephlebitis following cholecystectomy is an extremely rare occurrence, but it can happen due to a few possible mechanisms. During the laparoscopic cholecystectomy procedure, the pneumoperitoneum (carbon dioxide insufflation of the abdomen) can increase intra-abdominal pressure, leading to a decrease in venous flow and venous stasis. This stasis can create a favorable environment for bacterial overgrowth and subsequent thrombosis of the portal vein [7]. Another possible mechanism is the perforation of the gallbladder during the surgery, leading to the release of bacteria and bile into the peritoneum, which can spread to the portal venous system and cause thrombosis. Additionally, the manipulation and handling of the bile duct during the surgery can cause an iatrogenic injury, which can lead to bile leakage and subsequent infection of the portal venous system [7].

The pathogens most often found in pylephlebitis infections are *Bacteroides*, *Escherichia coli*, and *Streptococci *[7]. According to a survey of 19 cases by Plemmons et al. that covered the period from 1979 to 1993, diverticulitis was the most frequent cause, accounting for 13 instances (68%) [8]. The results of the same study revealed that bacteremia affected 88% of the patients and that *Bacteroides*, Gram-negative bacilli, and *Streptococci *were the most frequently identified microorganisms. In a retrospective assessment of the English-language literature from 1971 to 2009, 100 cases of pylephlebitis were found to have been documented [6].

The most common cause of pylephlebitis was found to be diverticulitis, which accounted for 30% of cases. Appendicitis (19%), inflammatory bowel disease (6%), and pancreatitis (5%) were also identified as frequent causes. Bacteremia was present in 60% of cases, with a single microbe found in 47% of cases and multiple bacterial organisms found in the rest [6]. No patients contained *Actinomyces*; the most prevalent pathogens were *Bacteroides*, *E. coli*, and *Streptococci*.

A total of 95 cases of pylephlebitis were discovered in a more recent retrospective analysis by Choudhry et al. that examined charts from 2002 to 2012. They investigated the reasons and discovered that pancreatitis, which accounted for 31% of the cases, was the most frequent cause, followed by diverticulitis (19%), peritonitis (15%), and intra-abdominal abscesses (13%). Noting that this research was conducted at a tertiary facility with a high volume of referrals for pancreatic and hepatobiliary disorders, which may not be representative of the general population. According to the same research, 34 individuals (44%) had bacteremia. *Streptococcus viridans*, *E. coli*, and *Bacteroides fragilis* were the most frequently grown microorganisms [7].

Pylephlebitis has nonspecific symptoms such as fever, abdominal pain, nausea, jaundice, and hepatomegaly, making it difficult to diagnose. According to Kanellopoulou et al., stomach discomfort (82%), fever (86%), and weariness (95% of all presenting symptoms), in that order, were the most frequent. According to the same research, leukocytosis (80%) was shown to be the most frequent in laboratory tests, followed by elevated liver enzymes (69%), hyperbilirubinemia (55%), and anemia (55%) [6]. Elevated levels of white blood cells, liver enzymes, bilirubin, and a decrease in red blood cells are commonly observed in laboratory tests for the patients. The diagnosis is usually confirmed by using imaging techniques such as Doppler ultrasonography or CT scans to detect portal vein thrombosis in patients with bacterial infections or intra-abdominal infections [9].

Effective management of pylephlebitis is crucial to avoid serious complications, and treatment mainly involves antibiotics. The choice of antibiotics should be based on the results of culture tests, and it is recommended to prescribe antibiotics for at least four weeks for patients without visualized abscesses and six weeks for patients with liver abscesses, with consideration of drainage [10]. Alternatively, a two-week parenteral antibiotic regimen followed by three to four weeks of oral treatment is another recommended approach.

The use of anticoagulation therapy in pylephlebitis remains controversial, with some suggesting that it could prevent thrombosis progression and related complications [11]. A retrospective study was conducted by Naymagon et al. on 67 patients with pylephlebitis to determine the effectiveness of anticoagulation. The study suggested that patients with pylephlebitis treated with anticoagulation have fewer chances of chronic portal hypertension with early resolution of portal vein thrombosis [11].

Different invasive techniques are available to treat pylephlebitis, including thrombectomy, catheter-directed thrombolysis, and systemic thrombolysis. For instance, Sherigar et al. reported a case where a patient was treated with alteplase after nine days of parenteral antibiotics with no improvement [9]. Surgical thrombectomy or percutaneous drain placement may be necessary in difficult cases, but surgical thrombectomy is associated with higher recurrence rates. Timely treatment is essential to prevent complications such as mesenteric ischemia, infarction, and extension of the thrombus to other veins, including the superior mesenteric vein and splenic vein [12]. Even with antibiotic treatment, the mortality rate of pylephlebitis is high at 11%-32% [3].

## Conclusions

Pylephlebitis is a rare but serious condition that can occur as a complication of intra-abdominal infections as well as after abdominal surgery as cholecystectomy in this case. In situations of fever and portal mesenteric venous thrombosis, it should be taken into consideration. The diagnosis of pylephlebitis can be challenging but bacteriemia and imaging studies such as Doppler ultrasonography or CT scan can aid in diagnosis. Treatment with antibiotics is essential to prevent complications, and the choice of early antibiotics should be based on culture results. Anticoagulation use should be considered for early resolution of portal vein thrombosis and prevention of chronic portal hypertension.
